# Management of Fibromyalgia: Novel Nutraceutical Therapies Beyond Traditional Pharmaceuticals

**DOI:** 10.3390/nu17030530

**Published:** 2025-01-31

**Authors:** Antonella Antonelli, Marzia Bianchi, Elizabeth Jane Fear, Luca Giorgi, Luigia Rossi

**Affiliations:** 1 Department of Biomolecular Sciences, University of Urbino, Cà Le Suore 2/4, 61029 Urbino, Italy; marzia.bianchi@uniurb.it (M.B.); luigia.rossi@uniurb.it (L.R.); 2 Department of Neuroscience, Imaging and Clinical Sciences, Institute for Advanced Biomedical Technologies, University “G. D’Annunzio” of Chieti-Pescara, 66100 Chieti, Italy; elizabeth.fear@unich.it; 3 Department of Pure and Applied Sciences, University of Urbino, Cà Le Suore 2/4, 61029 Urbino, Italy; luca.giorgi@uniurb.it

**Keywords:** fibromyalgia (FM), pain condition, diagnostic for FM, nutraceuticals, polyphenols, epigenetic regulation

## Abstract

The pathophysiology of fibromyalgia, a condition that causes chronic pain throughout the body, involves abnormal pain signaling, genetic predispositions, and abnormal neuroendocrine function, significantly impairing quality of life. Fibromyalgia is commonly characterized by musculoskeletal pain, chronic fatigue, and severe sleep alterations. Changes in the central processing of sensory input and defects in endogenous pain inhibition could be the basis of enhanced and persistent pain sensitivity in individuals with fibromyalgia. The term central sensitivity syndrome was chosen as an umbrella term for fibromyalgia and related illnesses, including myalgic encephalomyelitis/chronic fatigue syndrome, migraine, and irritable bowel syndrome. Given the substantial impact of fibromyalgia on health, there is a need for new prevention and treatment strategies, particularly those involving bioavailable nutraceuticals and/or phytochemicals. This approach is particularly important considering the adverse effects of current fibromyalgia pharmaceutical treatments, such as antidepressants and anticonvulsants, which can lead to physical dependence and tolerance. Natural products have recently been considered for the design of innovative analgesics and antinociceptive agents to manage fibromyalgia pain. Polyphenols show promise in the management of neuropathic pain and fibromyalgia, especially considering how anti-inflammatory treatments, including corticosteroids and nonsteroidal medical drugs, are effective only when inflammatory processes coexist and are not recommended as the primary treatment for fibromyalgia.

## 1. Introduction

**Figure 1:**
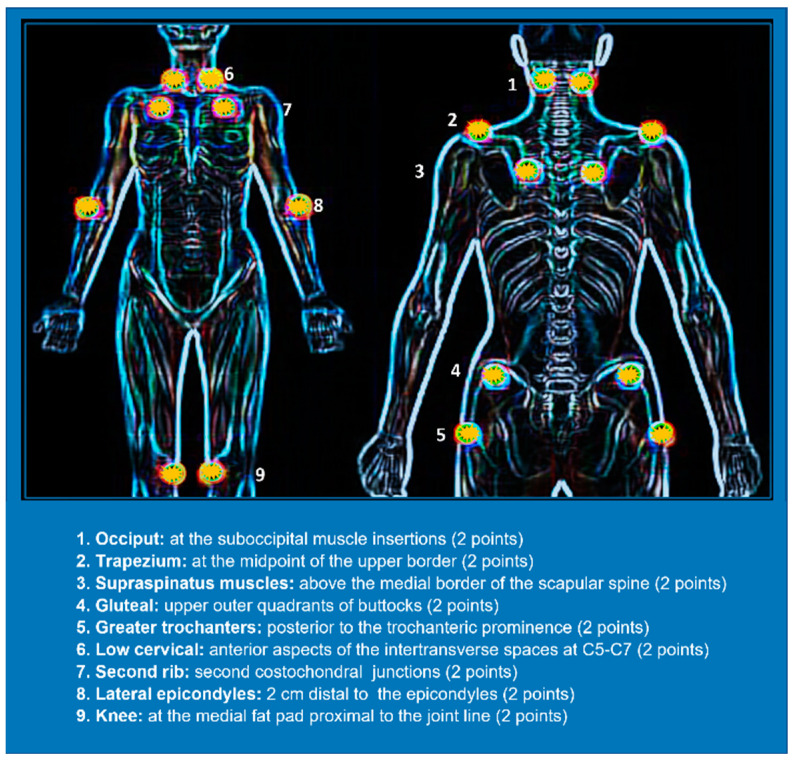
The orange spots correspond to the 18 trigger points used for fibromyalgia diagnosis.

Fibromyalgia (FM) is the second most frequent disease seen by rheumatologists, affecting more than 5% of the global population. While it primarily affects young and middle-aged women, FM can affect individuals of any gender or age who chronically suffer from widespread pain in the fibromuscular tissue, tendons, ligaments, and other areas. Although FM syndrome lacks a specific medical diagnosis, it is a condition that negatively affects the quality of life and involves sleep disturbances. Drugs used for the treatment of FM act by modifying the pain threshold, like those that can correct serotonin deficits and the neurovegetative hyperactivity present. The symptomatology may be triggered by several factors, such as hormone changes, physical injury, and intense emotional trauma. In fact, there are several studies that have clearly established that FM is influenced by abnormal neuroendocrine activity, genetic predispositions, and environmental triggers. In patients with FM, both the amplifying pain entity and constant widespread pain itself can be derived from alterations in the central nervous system and deficiencies in the body’s system that controls endogenic pain inhibition. The management of chronic pain in FM requires a varied medical plan that may include a combination of adjuvant medicines, psychological therapies, and behavioral strategies such as aerobic exercise to reduce distress and inflammation, as well as novel therapeutic strategies based on the use of nutraceuticals. FM typically results in pain that affects both sides of the body with distinct but specific “tender points” (points of hyperreactivity) in the ligaments, periosteal tissue, and muscles [1]. Tender points are the main sites that, if touched, produce pain and muscle spasms [2,3]. The literature has identified 18 allogenic points, with FM being revealed when pain is induced by digital pressure on at least 11 of these 18 points (Figure 1) [4]. A study by Schulze et al., involving both systematic control of published randomized trials and the efficacy of manual therapy for FM pain, demonstrated that myofascial release is the most commonly used intervention for patients with FM [5]. Researchers and clinicians are increasingly considering the recognition of a syndrome, without any causative disease or visible evidence of tissue damage, such as FM, as a pathological condition in which nociplastic pain results from altered nociception [6]. A clear definition of the conceptualization of pain as a disease is necessary, given the significant burden caused by this symptom. Researchers think that FM amplifies painful sensations by affecting the way the brain and spinal cord elaborate painful and non-painful signals.

## 2. Etiology

**Figure 2:**
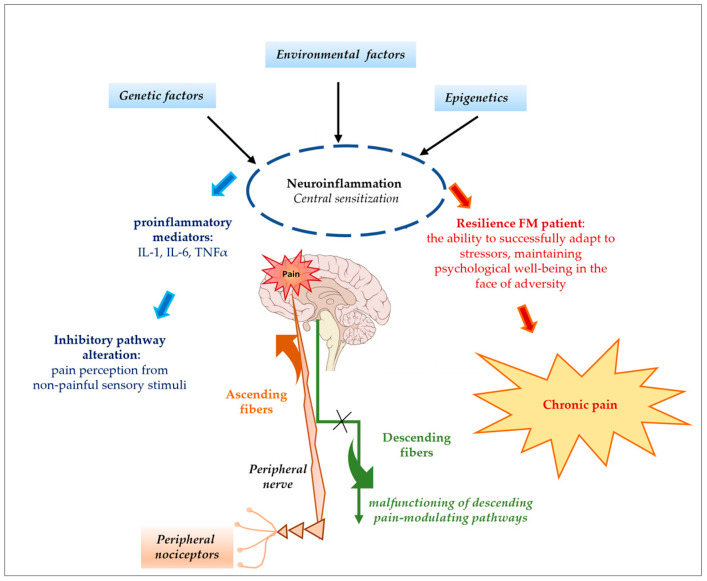
Ascending and descending pathways influence pain sensitivity. FM syndrome seems to depend on a reduced pain tolerance threshold due to an alteration in the perception modalities at the level of the central nervous system due to somatoaesthetic inputs (the alteration of the nociceptive threshold).

The etiology of dysfunctional pain in people with FM is multifactorial and includes both environmental (such as exposure to physical or psychological stressors) and genetic factors. Central sensitization and abnormal central nociceptive processing are two hallmarks of FM syndrome, which can now be classified as a neurosensory disorder (Figure 2). Patients with FM experience both enhanced pain sensitivity and the persistence of widespread pain, which appear to stem from changes in the central processing of sensory input and aberrations in the body’s natural pain inhibition mechanisms [7,8].

Imbalances between excitatory and inhibitory neurotransmission have been implicated in FM. Studies using magnetic resonance spectroscopy (MRS), more specifically proton (^1^H) spectroscopy in the brain, have identified altered neurotransmitter levels linked to these processes. Findings show elevated glutamate with increased glutamatergic activity in the insula and posterior cingulate cortex, suggesting that an overactive glutamate system is partly responsible for increased pain sensitivity [9]. Reduced gamma-aminobutyric acid (GABA) levels in the insula, indicating decreased GABAergic activity in this specific area of pain processing, has also been demonstrated [10]. Clinical studies based on functional magnetic resonance imaging (fMRI) have provided evidence of an alteration in nociceptive perceptions at the central neuronal level. Individuals with FM show intensified neuronal activation within pain-processing brain regions compared to healthy controls when subjected to the same amount of pressure stimuli [11].

Central sensitization occurs when the central nervous system becomes hypersensitive to pain signals, leading to the perception of pain even without a noxious stimulus [12]. This phenomenon is associated with areas such as the periaqueductal gray (PAG) and rostroven-tromedial areas of the descending pain pathway. These regions are mainly activated by opioid and serotonergic stimuli, respectively, and may act in endogenous analgesia. These two areas can modulate the transmission of nociceptive messages by connecting to the dorsal horn of the spinal cord. A neurochemical imbalance involving glutamatergic, 5-HTergic, and opioidergic systems is likely responsible for the changes found in these areas of the central nervous system, making these important targets for controlling this neurotransmitter balance. It is established that central sensitization and chronic oxidative stress are two fundamental mechanisms that have been identified as playing key roles in the development and maintenance of FM [13]. Erik A. Ovrom et al. have recently reviewed the possible genetic and epigenetic bases involved in FM development [14]. Polymorphisms that seem to affect the susceptibility and severity of the pathology have been found in genes implicated in the catecholaminergic and serotonergic pathways, pain processing, oxidative stress, and inflammation.

Sometimes, symptoms start after events such as physical trauma, surgery, infection, or important psychological stress. In other cases, symptoms have no single triggering event but gradually accumulate over time. Recent studies in the literature highlight that patients with FM express remarkably increased levels of miRNA-320a compared to controls [15,16], which drastically impact the severity of symptoms, like insomnia, chronic fatigue syndrome, persistent depressive disorder, and primary headaches, but are not related to the cerebral processing of pain. It appears that post-transcriptional regulation by microRNAs (miRNAs) may affect the expression of some proteins, exacerbating FM-associated symptoms [17,18,19,20,21,22,23,24,25,26,27,28,29,30,31,32]. Some miRNAs have been found to be differentially expressed in patients with FM versus healthy populations using multiplex approaches and referring to different compartments (cerebrospinal fluid, blood fractions, white blood cells, or serum), as summarized in Table 1. Jan L Bjersing et al., searching for a disease-specific pattern of cerebrospinal miRNAs in FM, identified nine microRNAs differentially expressed in patients with FM and healthy individuals [31]. Epigenetic insights into FM have even been extended to long noncoding (Lnc)RNAs, leading to the selection of two LncRNAs targeting most of the genes which are highly linked and present in the FM interactome, and which are responsible for establishing and developing the main features of this syndrome [33]. However, further studies are needed before these epigenetic markers can be of diagnostic and therapeutic relevance for FM.

Clearly, combined approaches in the field of clinical testing for FM may lead to more accurate diagnosis. Metabolic and proteomic profiling have also become important techniques in the analysis of potential biomarkers for FM. Wei-Hsiang Hsu et al. proposed a novel classification for FM based on pain and soreness symptoms, reporting data derived from metabolomic and proteomic profiling studies of urine and serum samples (from 30 patients with FM and 25 health control, HC). Significant differences in the expression of three metabolites in urine (hypoxanthine, diethylthiophosphate, and 4-guanidinobutanoic acid) and five metabolites in serum (tryptophan, isoleucine, SM(d18:1/18:0), PC(20:1(11Z)/18:0), and diethylthiophosphate), as well as eight proteins in serum (complement C1q C chain (C1qC), protein S100-A7 (S100A7), serpin B3 (SERPINB3), galectin 7 (LGALS7), lymphatic vessel endothelial hyaluronan receptor 1 (LYVE1), and fibrinogen alpha) were found in patients with FM in comparison to HC. This evidence is significant as it may provide insights into the pathological mechanisms of FM, leading to more effective diagnosis and therapy for FM. Moreover, these specific metabolites and proteins, which distinguish soreness and pain phenotypes in patients with FM, could serve as potential disease-relevant targets for developing subtype-specific treatments [34]. Other studies highlight how plasma metabolomics may be a useful comparative technique to investigate potential biological alterations in FM to better understand its biological mechanisms and phenotypic specificity, ultimately leading to the improved diagnosis of this disease [35]. Recent research has identified a group of small-molecular-weight proteins (S100) involved in inflammation and various cellular functions as a new area of interest in FM studies. Significantly increased levels of S100B and decreased levels of S100A7 were found in the serum of women with chronic pain conditions, including patients with FM, compared to healthy women. Therefore, S100 proteins could be potential targets for FM therapy and offer a novel strategy that considers the possibility of modulating the function of these selected proteins, potentially reversing the core symptoms of FM [36].

## 3. Evaluation of FM in Patients

In general, individuals with FM experience widespread pain in their whole body, including stiffness and aches in the joints and muscles, accompanied by unusual tiredness. To date, FM is still an “invisible disease”, that is, a pathological condition without visible symptoms [37]. Because of the wide variety of symptoms, FM blood tests focus on ruling out other conditions. Table 2 presents self-report forms used to evaluate patients’ pain, fatigue, and overall condition alongside a list of physical symptoms commonly associated with FM syndrome. A leading theory suggests that FM results from a brain malfunction that amplifies normal nerve responses, causing FM individuals pain or other symptoms without any apparent triggers [7].

Despite the serious effects of FM on quality of life and its recognition by the World Health Organization as a disease since 1992, it has not yet been officially recognized as a chronic disabling disease in all countries. It is still not listed in the EU’s official diseases registry or recognized by the national health services in many Member States, although an estimated 19% of Europeans are statistically affected (https://www.europarl.europa.eu/doceo/document/E-9-2021-003391_EN.html (accessed on 30 June 2021)). Regarding FM as a “disease-non-disease” has greatly limited the scope and number of clinical studies aimed at demonstrating the effectiveness of various therapeutic approaches. Consequently, there are no universally accepted guidelines for the clinical and therapeutic management of patients. Since the symptoms are like other conditions, doctors typically aim to rule out illnesses such as arthritis, lupus, ankylosing spondylitis, polymyalgia rheumatica, Sjogren’s syndrome, underactive thyroid or hypothyroidism, endocrine disorders, connective tissue problems, neurologic conditions, muscle inflammation, or other diseases. The European League Against Rheumatism (EULAR) recommendations are accompanied by high-quality reviews and meta-analyses aimed at identifying research priorities, clarifying who will benefit from specific treatments, and establishing evidence-based criteria for the use of individual treatments, whether pharmacological or non-pharmacological and how these treatments can be effectively combined [38]. The EULAR recommendations include drugs such as analgesics, skeletal muscle relaxants, antidepressants, anticonvulsants, alpha-2 agonists, and more, which are reported in Table 3.

Firstly, it is important to highlight the importance of routinely monitoring parameters such as those reported in Table 4. These measurements can provide valuable insight even though patients with FM do not typically display characteristic or consistent abnormalities in laboratory tests [39]. Additionally, imaging studies can help exclude pathologies with similar symptoms and assist in the diagnosis of some inflammatory diseases that often coexist with FM. The international literature highlights the significant challenges in diagnosing FM in the absence of observable signs. Despite this, FM is a real pathology though it has historically led to patients being classified as “psychosomatic”, anxious or even “imaginary patients”. The recommended laboratory tests are limited to an initial evaluation via complete blood count and PCR, since FM is not an inflammatory condition. In practice, all evaluations are necessary for making a differential diagnosis, aiming to exclude other potential conditions such as peripheral neuropathy, systemic lupus erythematosus, spondylarthritis, connective tissue diseases, and hypo-hyperparathyroidism. FM is defined as primary, or idiopathic, when it is not associated with another pathology. It is defined as secondary when diagnosed with other clinical conditions as a typically chronic disease. FM ranks second only to osteoarthritis among musculoskeletal disorders. Patients report feeling more fatigued upon waking in the morning than they did the previous evening. The pain is accompanied by painful muscular contractions in both girdles and the paravertebral area, which tend to be more intense upon waking.

**Table 3:** Medications prescribed for the management of FM. Notes: * the only drugs officially approved by the US Food and Drug Administration (FDA) for the treatment of fibromyalgia.

Examples of Drugs for Patients with FM Condition	
Tramadol (ULTRAM)	synthetic opioid painkiller [38,40,41]
Amitriptyline (ELAVIL)	tricyclic antidepressants [37,42,43]
Pregabalin * (LYRICA) Gabapentin (NEURONTIN)	drugs for neuropathic pain [37,43,44]
Duloxetine * (CYMBALTA) Milnacipran * (SAVELLA)	serotonin and norepinephrine reuptake inhibitors (SNRIs) [37,38,39,40,41,45]
Cyclobenzaprine (FLEXERIL, FLEXMID)	muscle relaxants by blocking nerve impulses that are sent to the brain [40]
Naltrexone (NALOREX)	a low dose may work as an anti-inflammatory agent in the central nervous system [46]
Tizanidine (ZANAFLEX)	alpha-2 adrenergic agonists helping to relieve muscle spasms and easing pain [41,44]

**Table 4:** Laboratory testing useful for patients with FM.

***Complete Blood Count (CBC) with a differential test****:* measuring the total number of blood cells and breakdown of each type of white blood cell.
***Metabolic panel:*** this test provides important information about metabolism and the balance of certain molecules in the body (e.g., glucose, calcium, sodium, potassium, bicarbonate, chloride, albumin, total protein, alkaline phosphatase, alanine transaminase, and aspartate aminotransferase, bilirubin, creatinine).
***Urinalysis test:*** this test assesses urinary metabolites. Taurine, creatine, and succinic acid are essential biological markers of the pain and fatigue symptoms in patients with FM.
***Thyroid-stimulating hormone levels:*** determining these levels indicates prevalence and a relationship in patients with FM with acute thyroiditis and/or thyroid autoimmunity disease.
***25-hydroxy vitamin D level:*** some studies report an association between vitamin D deficiency and fibromyalgia. The role of this vitamin in the pathophysiology of FM and its clinical relevance requires further elucidation with appropriately controlled studies.
***Vitamin B12 level:*** vitamin B12 deficiency can be associated with relevant fatigue and prespecified neurologic symptoms in patients with FM.
***Iron studies:*** these include the iron level, total iron binding capacity, percent saturation, and serum ferritin level.
***Magnesium level:*** the benefit of magnesium administration in patients with FM has been suggested by different studies.
***Erythrocyte sedimentation rate (ESR) test:*** serum levels of inflammatory markers, including ESR, CRP, NLR, and MPV, are higher in patients than in controls, which makes them of good diagnostic value in patients with fibromyalgia.
***Antipolymer antibody assay:*** this test provides evidence for a subgroup of people with FM; it is found that about 50% of patients with FM have antipolymer antibodies.

## 4. Management with Pharmaceuticals

### 4.1. Overview

The prompt diagnosis and treatment of FM can substantially improve the quality of life for patients. The treatment plan should focus on three fundamental aspects: (1) pain relief; (2) physical therapy to fortify the body; and (3) addressing the emotional and psychosomatic components of the disease. Treating pain as a pathophysiological condition can increase recognition of this worldwide health problem, and new treatment possibilities can be explored, especially as the prevalence of these pain syndromes is increasing. The US Food and Drug Administration (FDA) has approved three drugs for use in FM, including pregabalin (Lyrica), duloxetine (Cymbalta), and milnacipran (Savella). Moreover, other medications, such as amitriptyline, cyclobenzaprine, gabapentin, and tiagabine, are usually considered first-line treatments [40]. Each of these medications was originally prescribed for other conditions, such as depression or nerve pain. Pregabalin is used to reduce pain and improve sleep. Milnacipran and duloxetine have shown some efficacy on some clinical parameters of FM in patients (referred pain, musculoskeletal stiffness, and asthenia) in some randomized clinical trials [41]. Pregabalin has been shown to be beneficial in relieving pain and sleep disturbances in FM but not asthenia and mood changes [44]. The antidepressants duloxetine and milnacipran, generally used for the treatment of depression, can be administered at lower doses to relieve pain, fatigue, and sleep disturbances in FM cases. Muscle relaxants, such as cyclobenazaprine (Flexeril), can help treat muscle spasms. However, these three FDA-approved drugs for FM are quite expensive, and their efficacy varies greatly among patients. Only in cases of coexisting inflammatory processes can corticosteroids and anti-inflammatory drugs be administered, but not as a first-line intervention strategy. Some studies have reported potential therapeutic approaches using a reserpine-induced myalgia animal model (rats and mice), which mimics nociplastic pain [47]. The repeated administration of reserpine depletes endogenous monoamines, disrupting monoaminergic control in the central nervous system. This results in a marked decrease in dopamine, noradrenaline, and serotonin levels in the brain, inducing muscle hyperalgesia and mechanical allodynia. These symptoms last for weeks without any apparent organic abnormalities, thereby mimicking nociplastic pain in patients with FM. This model highlights the biologic processes behind nociplastic pain, such as reduced monoamines, increased oxidative stress, inflammation, and sensitized sensory nerves. It can be concluded that potential treatments for nociplastic pain include targeting mediators responsible for chronic inflammatory pain and neuropathic pain, correcting nutritional imbalances using nutritional supplements and natural substances to improve the physical constitution, and using non-pharmacological interventions [48].

### 4.2. Analgesics

Nonsteroidal anti-inflammatory drugs (NSAIDs) are commonly prescribed to manage pain in FM despite evidence from clinical studies showing that their effectiveness in treating this condition is extremely limited, if not nonexistent.

NSAIDs inhibit the production of prostaglandins and, thus, can lessen the peripheral and central sensory hypersensitivity that occurs with nerve injury-associated inflammation. Sheena Derry et al. [49] published an Intervention Review in 2017 examining data from trials involving oral nonsteroidal anti-inflammatory drugs, such as etoricoxib, naproxen, and tenoxicam, for the treatment of FM in adults. Some NSAIDs may alleviate pain by blocking particular enzymes that are part of the body’s response to painful stimuli [50]. However, it is not clear whether this mechanism is effective against the unusual types of pain associated with FM. For this reason, the trend with healthcare providers is to prescribe fewer NSAIDs and instead prescribe drugs that work on the central nervous system, which is where the pain is believed to stem from [49]. In addition, several types of NSAIDs (such as ibuprofen, ketoprofen, diclofenac, naproxen, and aspirin) that are available over the counter each have their own dangers and possible side effects. Moreover, these drugs may lessen pain, but they are not effective for everyone.

### 4.3. Antidepressants and Anticonvulsants

Paradoxically, although amitriptyline has not been formally approved for FM treatment, numerous patients have reported experiencing relief with its use; for example, it is accepted that low doses of tricyclic antidepressants, such as amitriptyline (Elavil), may help treat sleep disturbances. Amitriptyline, which was initially developed as a tricyclic antidepressant, has been used in the management of pervasive pain and the exhaustion characteristic of FM. It treats neuropathic pain, increasing serotonin levels in the brain and reducing nerve sensitivity. By increasing serotonin levels, amitriptyline is believed to strengthen the body’s own pain regulation mechanisms. This action is beneficial for reducing the specific type of pain that patients with FM experience. Pregabalin (LIRYCA) is currently used to treat FM. Pregabalin is an anticonvulsant belonging to a group of medicines used to treat a type of epilepsy (partial seizures with or without secondary generalization) in adults. Pregabalin is used to treat long-lasting pain caused by nerve damage. Neuropathic pain can be caused by FM and other different types of diseases. The pain sensations may be described as hot, burning, throbbing, shooting, stabbing, sharp, cramping, aching, pricking, numbness, or tingling. Central and peripheral neuropathic pain may also be associated with mood changes, sleep disturbances, and fatigue (tiredness) and may impact physical and social activities and overall quality of life. Pregabalin is used to treat Generalized Anxiety Disorder (GAD), characterized by excessive and prolonged anxiety and worry that is difficult to control [44,51]. GAD can also cause restlessness or a feeling of being agitated. Pregabalin can interact with certain molecules in the brain to counteract seizures, nerve pain, and anxiety however the exact mechanism is not yet known. Medications designed to treat epilepsy are often useful in reducing certain types of pain. Gabapentin (Neurontin) is sometimes helpful in reducing fibromyalgia symptoms, while pregabalin (Lyrica) was the first drug approved by the Food and Drug Administration to treat fibromyalgia (Table 3).

### 4.4. Other Medications

Various treatment options currently exist that help alleviate symptoms. Neuro-modulatory agents have shown the potential to positively influence the symptoms of patients with FM. Alongside medications such as gabapentinoids (e.g., pregabalin) and serotonin–norepinephrine reuptake inhibitors (e.g., duloxetine), other therapies are used for the treatment of FM. These include the administration of cannabinoids (e.g., Dronabinol), although only small and limited studies have been conducted to date, as well as opioids (e.g., Tramadol, Buprenorphine, Tapentadol). Doctors may prescribe Tramadol (Ultram), which is often used to treat FM pain. Tramadol is a synthetic drug belonging to a class of opioid painkillers. It is used in the treatment of painful conditions, in particular, to manage chronic pain. It is a drug capable of interacting with opioid receptors and is also able to inhibit the reuptake of noradrenaline and serotonin. Used at low doses, it seems to have a beneficial effect on pain in the absence of substantial adverse effects. Naltrexone at low doses (4.5 mg oral daily for 12 weeks), in addition to antagonizing opioid receptors at the neuronal level, can inhibit microglia cell activity and invert central and peripheral inflammation. It was also found to reduce FM symptoms in an entire cohort, with a reduction greater than 30% compared to the placebo. Furthermore, the threshold of thermal pain and mechanical pain is improved using this drug [42,43,46]. Among other medications tested in FM, quetiapine, a second-generation atypical antipsychotic drug, has attracted interest in the treatment of chronic pain, including FM [52].

Furthermore, there are also non-pharmacological interventions like acupuncture, electric stimulation, vibroacoustic and rhythmic sensory stimulation, thermal therapies, hyperbaric treatments, laser therapy, phototherapy, cryotherapy, osteopathic manipulative techniques, as well as probiotics, and the use of plant extracts and natural molecules [53]. Certain treatment solutions involve psychological aspects of the disease, like self-management skills and cognitive behavioral therapy, while other solutions are developed to strengthen the body by including physical activities and myofascial physical therapy, thus reducing pain [54,55]. Among the current solutions, it is important to note that many potential treatments for FM, particularly those addressing chronic pain, have not yet progressed significantly to human clinical trials. Additionally, in clinical trials or pilot studies, the heterogeneity of patients based on FM diagnostic criteria can present challenges in the evaluation of clinically meaningful effects of treatments. Furthermore, a patient’s adherence or abandonment of the treatment must also be considered [56,57]. The literature reports preclinical studies indicating the potential use of some natural compounds with antinociceptive activity, such as phenolic compounds (e.g., flavonoids) from medicinal plants, and these are reported in the next section of this paper (5. Management with natural products and nutraceuticals). These studies show promising results as support in FM therapy. However, further research with rigorous methodology is needed in order to investigate these treatments’ efficacy and safety.

## 5. Management with Natural Products and Nutraceuticals

### 5.1. Overview and Potential Mechanisms of Action

Pharmaceutical drugs available today to treat FM have adverse effects, including physical dependence and tolerance. Therefore, the great availability of nutraceuticals has drawn considerable attention from patients seeking relief from chronic pain conditions.

The potential mechanisms of action of the many natural molecules cited here have already been reported in our previous article [58], where we extensively described several molecular pathways, including those related to oxidative stress and its association with fatigue in FM. These mechanisms are specifically reported in Table 2, which details various biological activities, such as the antioxidant and anti-inflammatory nature of polyphenols, as well as their ability to modulate molecular targets. In particular, the MAPK signaling pathway, which induces the inflammation process, stimulates microglial activation and pain sensitization via many signaling molecules, such as chemokines, cytokines, and kinases regulated by the p38 protein. Phosphorylated p38 activates NF-kB, which triggers the release of additional inflammatory mediators, including cyclooxygenase-2 (COX-2), the brain-derived neurotrophic factor (BDNF), IL-6, TNF-α, and IL-1β. These mediators cause the sensitization of the central nervous system to pain signals and stimulate the development of FM syndrome.

### 5.2. Palmitoylethanolamide (PEA)

Selected novel drugs for FM, such as PEA, an endogenous endocannabinoid mediator, commercialized as a dietary food for special medical purposes, down-modulates the activation of mast cells and microglia and has anti-inflammatory and antihyperalgesic properties, involving the activation of nuclear peroxisome proliferator-activated receptor-α (PPAR-α) and the orphan G-protein coupling receptor (GPR55). In conditions of neuropathic pain, levels of PEA in brain regions involved in nociception have been reported to be altered [59]. PEA, as a fatty acid amide from the group of N-acetylethanolamides, does not bind to classical cannabinoid receptors, but it indirectly stimulates endocannabinoids. The reduction in pain and improvement in quality of life following PEA treatment aligns with the involvement of microglia and mast cells in FM and their association with the presence of pain [45]. A recent randomized placebo-controlled study on healthy volunteers highlighted that PEA acts by reducing peripheral and central sensitization while also increasing pain modulation [60]. A new generation of nutraceuticals has been formulated, with synergistic action, as a useful adjuvant in managing FM symptoms. This innovative formula rich in PEA, Alpha-Lipoic Acid (ALA), and *Gynostemma Pentaphyllum* is specifically designed for the treatment of FM symptoms. The synergistic combination of PEA, ALA, and *Gynostemma Pentaphyllum* with high bioavailability helps neural and synaptic trophism, reduces oxidative stress, and combats the symptoms of asthenia.

### 5.3. Capsaicin

Notably, capsaicin, an alkaloid abundant in the fruits of *Capsicum* species or hot chili peppers, acts as an analgesic in diabetic neuropathy and chronic musculoskeletal pain, as well as having cardioprotective and anti-inflammatory effects. This alkaloid molecule alleviates pain associated with several types of neuropathic and musculoskeletal pain in different ways, such as inhibiting the synthesis of pro-inflammatory mediators (IL-1β, IL-6, TNF-α), which aggravate hyperalgesia and allodynia [58].

### 5.4. Ashwagandha and Withania somnifera

Also worth mentioning is the root of Ashwagandha, which belongs to the Solanaceae family, and whose extract has not only powerful anxiolytic and cognitive enhancing effects but also has a powerful anti-inflammatory and analgesic efficacy due to its direct action on nociceptive nerves [61,62]. Among steroidal lactones, called *withanolides*, contained in the Ashwagandha (or *Withania somnifera*), withaferin-A, withanolide-D, and withanone are the most important ones responsible for bioefficacy showing diverse pharmacological activities [63]. They are potent antioxidants able to quench-free radicals and other reactive oxygen species inhibiting free-radical-induced cell damage. Through the upregulation and downregulation of some transcription factors that control the production of regulatory macromolecules, these bioactive molecules control the expression of various enzymes, receptors, and other regulatory proteins that are involved in the pathogenesis of various diseases [64]. Withaferin-A is present in high concentrations in *W. somnifera* plant extracts and interacts with the effectors of multiple signaling pathways involved in inflammatory response, oxidative stress response, cell cycle regulation, and synaptic transmission [65].

### 5.5. Curcumin

The main polyphenol of turmeric rhizome, *Curcumin*, has shown clear analgesic effects in several pathologies involving chronic conditions (like rheumatoid arthritis, inflammatory bowel disease, Alzheimer’s disease, and common malignancies like colorectal cancer) even if long-term efficacy in musculoskeletal pain remains to be defined, due to the lack of randomized clinical studies in the field [66,67]. By using animal models, some authors have demonstrated that curcumin, with its potential anti-inflammatory and antioxidant effects, is able to suppress numerous cell-signaling pathways, including the nuclear factor kappa-light-chain-enhancer of activated B cells (NF-κB), the signal transducer and activator of transcription 3 (STAT3), nuclear factor-erythroid factor 2-related factor 2 (Nrf2), ROS, COX-2, superoxide leukocyte recruitment, and oxidative stress [68]. It has already been reported that curcumin can prevent tumor growth, angiogenesis, epithelial–mesenchymal transition, invasion, and metastasis by modulating the expression of tumor-related noncoding RNA (ncRNA). It is now well known that gut dysbiosis is the primary cause of the initiation and development of many chronic diseases. Recent reports also suggest curcumin plays a crucial role in regulating the gut microbiota via the biotransformation of curcumin and its metabolites. In addition, low bioavailability issues appear to be overcome by including bioavailability-enhancing compounds such as *Piperine* in *Curcumin* formulations [69]. Moreover, curcumin seems to have important effects on the central nervous system, improving cognitive functions and attention in people affected by post-traumatic stress syndrome. Interestingly, a study reported the use of red blood cell membrane (RBCM)-mimicking liposomes containing curcumin (named RC-Lip) for the treatment of diabetic wounds, showing a prospective strategy of efficiently mediating the inflammatory response. RC-Lips were successfully fabricated using the thin film dispersion method, and the fusion of the RBC membrane with the liposomal membrane was confirmed via surface protein analysis. The use of this approach, along with similar methods that involve mimicking red blood cells (RBCs) or erythrocytes for substance delivery, has already been reported in the literature. It is to be noted that the natural properties of RBCs make them potential candidates for use as drug carriers or for camouflaging compounds due to their innate biocompatibility. They have gained attention for their unique ability to retain properties post-manipulation, which helps them remain non-immunogenic in the biological environment, thereby prolonging their circulation time and improving therapeutic efficiency [70,71,72,73]. It is well known that drug delivery using natural biological carriers, especially RBCs, is a rapidly growing field since these cells can act as carriers that are able to prolong the drug’s action due to its gradual release from the carrier itself. Some authors have recently suggested that encapsulating certain terpene indole alkaloids such as vincristine and vinblastine isolated from the plant *Catharanthus roseus G. Don* (*Vinca rosea Linn.*) into RBCs could partially solve the problem of side effects and toxicity by reducing the peak concentration of drugs in the bloodstream [74]. Recently, Fereshteh Safarpour et al. [74] described an inspiring biomimetic system based on the RBCM vesicles for improved encapsulation efficiency and the release of curcumin (Cur). In this work, the role of the sonication time on the properties of RBCM-Cur vesicles was studied, showing that the hydrodynamic vesicle size, zeta potential, and release behavior are adaptable by changing the sonication time. Furthermore, it was observed that the entrapment efficiency of Cur polyphenol, used as a model drug, remained high throughout the entire sonication process due to the amphiphilic nature of RBCM. The authors described the use of biological vesicles based on the RBCM as drug delivery systems of hydrophobic drugs such as Cur and also investigated its release ability [75,76].

### 5.6. Coenzyme Q10

In terms of nutritional therapy for FM, the administration of dietary supplements to support the treatment of this condition is receiving increasing scientific and clinical recognition. The use of specific supplements for FM is based on the idea that the pathogenesis of FM depends, at least in part, on an alteration in energy metabolism at the muscular level associated with a deficiency of Coenzyme Q10 and B vitamins [77]. In patients with FM, muscular energy dysfunction could produce generalized oxidative stress [78], which is capable of inducing a systemic inflammatory state and altering the function of peripheral nerves and the central nervous system, giving rise to the entire spectrum of symptoms in FM syndrome, including neuropsychiatric ones (anxiety, depression, drowsiness, difficulty in concentrating) [79,80]. Improving muscle energy function could, therefore, impact not only pain and asthenia but also central symptoms. Coenzyme Q10 (ubiquinone) is present in the mitochondria of all cells and is involved in all phases of aerobic energy production and participates in many oxidoreductive reactions (therefore assuming a strong antioxidant capacity); the concentration of Coenzyme Q10 at the muscle level tends to decrease with increasing age. The dietary supplementation of Coenzyme Q10 has been successfully tested in patients with FM in numerous studies. By improving muscle metabolic parameters, the intake of Coenzyme Q10 can reduce pain and asthenia. The improvement in depressive symptoms after Coenzyme Q10 supplementation seems to be linked to the normalization of serotonin levels in the central nervous system [81,82]. The combined supplementation of Coenzyme Q10, B vitamins (B12, B6, B1), folic acid, and carnitine should have superior and synergistic effects compared to individual nutrient supplementation, resulting in an even greater clinical efficacy in the treatment of FM.

### 5.7. Alpha-Lipoic Acid

Another nutrient with antioxidant and trophic action for the nervous system is Alpha-Lipoic Acid (ALA), which has been shown to be effective in reducing musculoskeletal pain in patients with FM [83]. ALA, also known as “thioctic acid”, is a sulfur compound produced primarily in the liver and is found in tissues with high metabolic activity, such as the heart, liver, kidneys, and brain. ALA is an important enzymatic cofactor and participates in numerous cellular processes responsible for the production of ATP (energy), mainly in mitochondria as well as oxidation–reduction reactions. ALA is involved in regenerating other endogenous redox substances, such as Coenzyme Q10, vitamin C, and vitamin E, enabling them to continue their vital role in combating free radicals. ALA levels decrease significantly with age, making it an ideal dietary additive. Supplementing ALA has proven effective in reducing pain in patients with FM. Since ALA has shown promise in patients with neuropathic pain, which has similar features and symptoms to FM, several trials have investigated the safety and efficacy of this antioxidant for the treatment of FM pain, with the aim of understanding if ALA can effectively provide benefit in FM [84,85].

### 5.8. Carnitine

Carnitine is another essential nutrient crucial for proper neuromuscular function. It plays a key role in oxidative metabolism because it facilitates the transport of fatty acids into the mitochondria, where they are used to produce ATP. In patients with FM, low levels of carnitine have been found in muscle tissue [86]. Carnitine, derived from the methylation of the amino acid lysine, is vital in the metabolism of fatty acids, with the carnitine palmitoyl transferase (CPT I) system controlling fatty acid oxidation. Skeletal and cardiac muscles expressing CPT I use fatty acids as their primary source of energy. In fact, carnitine deficiency is often associated with low energy levels, muscle weakness, and general fatigue [87]. Randomized clinical studies have shown that dietary supplementation of carnitine reduces skeletal muscle pain and depressive symptoms in FM. One study showed that the effects of carnitine on pain and depression were comparable to those obtained with the antidepressant duloxetine [88]. Therefore, carnitine may not only provide support for the muscular system of patients with FM but also contribute to general and mental health benefits [89].

### 5.9. Vitamins

#### 5.9.1. B Vitamins

A few studies suggest that low vitamin B12 (B12) levels may be involved in FM as well as in chronic fatigue syndrome and it is possible that they are, at least in part, responsible for the low energy levels typical of both conditions. The supplementation of vitamins important for proper muscle and nerve function has also been shown to be clinically effective in FM. The first clinical trial to assess the association between B12 deficiency and the prevalence of fatigue and prespecified neurologic symptoms in patients with FM has been published [90]. Other researchers have focused on the efficacy of a daily oral dose of 1000 mcg of vitamin B12 on the symptom severity and psychological profile of patients with FM. The study found improvements in disease severity and anxiety in patients with FM, with no reported side effects at the dosage studied. Based on the results, the authors suggested that vitamin B12 has strong potential as an adjunctive therapy for FM [91]. Some patients with FM have clear deficiencies of vitamin B12 and folic acid (B9); in these patients, the dietary supplementation of cobalamin (B12) and folates has significantly reduced the use of painkillers and the feeling of asthenia. The supplementation of other B vitamins (B1, B6) has also been shown to be beneficial in FM [92]. It has also been recognized that the combined supplementation of Coenzyme Q10, B vitamins (B12, B6, B1), folic acid, and carnitine should have superior and synergistic effects compared to the supplementation of the individual nutrients, resulting in even greater clinical efficacy in the treatment of FM.

#### 5.9.2. Antioxidant Vitamins

The oxidative stress component is very important in the pathogenesis of FM. Studies have shown that patients with FM often show a reduced function of endogenous antioxidant enzymes [93] and low levels of antioxidant vitamins [94]. Increased levels of prooxidative factors such as nitric oxide and lipid peroxidation can cause pain sensitization in FM. As a result, nutrients with strong antioxidant functions, including vitamin C, vitamin E, and vitamin A, could have a beneficial role in FM. Some research also discusses the impact of various antioxidative procedures, such as modification to dietary habits, which can diminish FM symptoms [84,95].

#### 5.9.3. Vitamin D

FM has been linked to vitamin D deficiency, especially in postmenopausal women [96].

Some authors have reported meta-analysis studies showing that the vitamin D serum levels of patients with FM were significantly lower than those in the control group, suggesting that this vitamin can be a key factor in FM. Therefore, vitamin D supplementation among women appears to be a potential preventive strategy [97,98].

Several clinical studies have evaluated the effect of vitamin D supplements on musculoskeletal pain: vitamin D significantly reduces pain but does not show beneficial effects on associated psychopathological symptoms. Preclinical and recent clinical studies relating to the use of most of the previously described supplements and their molecular mechanisms have already been reported [99,100].

### 5.10. Minerals

#### 5.10.1. Magnesium

Magnesium (Mg) has also been shown to reduce inflammation and pain associated with FM symptoms, playing a critical role in muscle relaxation and neurotransmitter function. The effectiveness of magnesium supplementation lies in its ability to prevent the development of typical central nervous sensitization both centrally and peripherally in FM individuals. Low Mg levels are commonly found in patients with FM, and Mg deficiency can reduce exercise capacity, increase inflammation [101], and contribute to muscle spasms. This is believed to be due to the role of Mg in the regulation of muscle function and adenosine triphosphate (ATP) production. Mg is essential for ATP synthesis because ATP is stored in the body as a magnesium–ATP compound. Low ATP levels are also common in people with FM, and this is thought to play a significant role in the development of many of the condition’s symptoms [102]. Furthermore, it has been reported that Mg is also involved in the regulation of hormone synthesis, including norepinephrine, which is often overproduced in patients with FM. This hormonal dysregulation is believed to contribute to the pathogenesis of FM. In addition, Mg is involved in the regulation of various nerve receptors, such as N-methyl-d-aspartate receptors, which are involved in the development of neuropathic pain. Magnesium’s ability to block these receptors may help alleviate certain FM symptoms [103].

#### 5.10.2. Iron

In this context, the role of another important element, iron (Fe), has been shown. Fe is essential for many biosynthesis mechanisms, including neurotransmitter synthesis. Low Fe levels (and decreased ferritin) in the body can lead to a reduced synthesis of biological amines, which is considered a pathological mechanism in patients with FM [104]. Data obtained from a nationwide population-based cohort study [105,106] demonstrated that iron deficiency anemia is often associated with an increased risk of FM and that the overall incidence of FM is higher in individuals with this type of anemia compared to the non-iron deficiency anemia controls. Current results also suggest that ferric carboxymaltose, which is well tolerated, offers benefits for iron-deficient patients with coexisting FM, showing improvement in FM severity when compared to a placebo [107].

### 5.11. Carotenoids

Recently, the antinociceptive, antioxidant, and antidepressant effects of cationic liposomal carotenoids at the nanoscale have been investigated in the reserpine model of FM. For this purpose, carotenoids were formulated as beta-carotene-encapsulated cationic liposomes (CL-Bc) and lutein-encapsulated cationic liposomes (CL-Lut) and characterized. Monoamine levels and oxidative stress markers were measured in the cortical tissues of female rats. Furthermore, electrocorticogram (ECoG) spectral analysis and behavior testing were conducted. After two weeks of cationic liposome carotenoid treatment, all the measured parameters showed a return to the control values with no significant difference. The results provide evidence that natural product preparations such as cationic liposomal carotenoids could be a valuable alternative to the pharmacological treatment of FM [48]. Among the vast number of natural dietary supplements that are used to alleviate neuropathic pain as well as FM symptoms, polyphenols are of particular interest [100,108].

### 5.12. Polyphenols

Currently, while the regular consumption of dietary polyphenols is likely to be beneficial for human health [109], it is important to note that they are extensively metabolized in the digestive tract or other parts of the body before reaching the target organs. As a result, significant research has been conducted on developing new delivery systems for natural molecules to ensure that polyphenols are transported to organs and tissues in a favorable form [110]. Ideally, like rheumatic diseases, where evidence suggests that the regular consumption of polyphenols can have therapeutic effects in relieving symptoms, data from randomized controlled trials on the administration of polyphenols and their effects on FM should also be collected. These trials should assess the relationship between polyphenol administration and the mitigation of FM syndrome in humans [111]. Strategies based on the use of natural active molecules, either alongside or as an alternative to pharmacological treatment, could offer the best approach for managing patients with FM. This is particularly relevant since drug therapies used to alleviate symptoms do not address the underlying causes of the pathology itself and are often poorly tolerated by individuals with this chronic pain. It is important to note that treating FM requires a comprehensive, multidisciplinary approach. This includes a combination of drugs and natural treatments (like polyphenols and other natural active molecules), along with lifestyle changes, including good nutrition, vitamin supplementation, weight loss, regular physical exercise as well as specific physiotherapy aimed at improving symptoms and reducing pain in patients with FM.

Plant-based diets are increasingly recognized by nutritionists as effective in managing the pain of FM in patients; in recent years, nutritional interventions have gained significant interest in the field of FM treatment despite the lack of clear dietary guidelines to optimize the management of FM in patients. This review highlights the potential of plant-based nutraceuticals as a dietary intervention to reduce inflammation associated with this syndrome and improve the quality of life for patients with FM.

**Figure 3:**
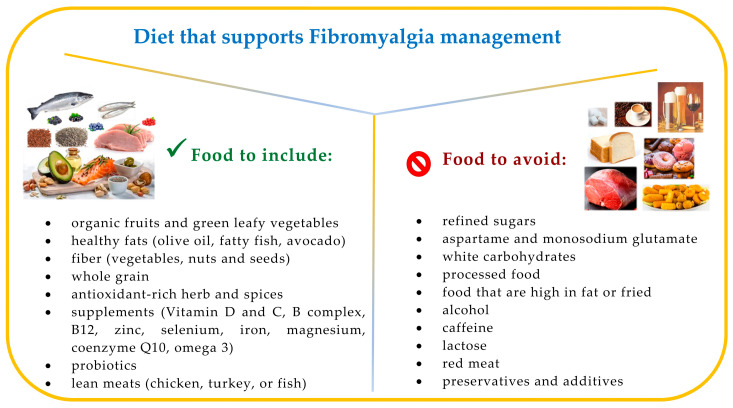
Fibromyalgia: recommended foods and foods to avoid.

Although nutrition alone cannot cure fibromyalgia, it can help alleviate some of the symptoms. It is possible to exploit the nutritional characteristics of different foods to improve the individual’s health. Systematic reviews of the literature have been conducted to assess if diet affects widespread pain and gastrointestinal disorders for individuals with FM. They also summarized future research directions [103,112]. There is no specific diet that is effective for all patients; however, it is important to emphasize that patients seem to derive numerous benefits from an improvement in their eating habits. A proper diet rich in foods with anti-inflammatory properties, including red fruits, leafy vegetables, nuts, and seeds, as well as fatty fish, appears to play a crucial role in FM management (Figure 3).

Some studies have shown that natural products have an analgesic effect in various animal models of FM, likely by activating inhibitory descending pathways such as the periaqueductal gray (PAG) and rostroventromedial medulla. Natural products and their secondary metabolites could, therefore, be a promising source for FM management [113]. However, there are limited numbers of translational studies to validate the potential mechanisms of nutraceuticals used in the treatment of neuropathic pain and FM.

**Figure 4:**
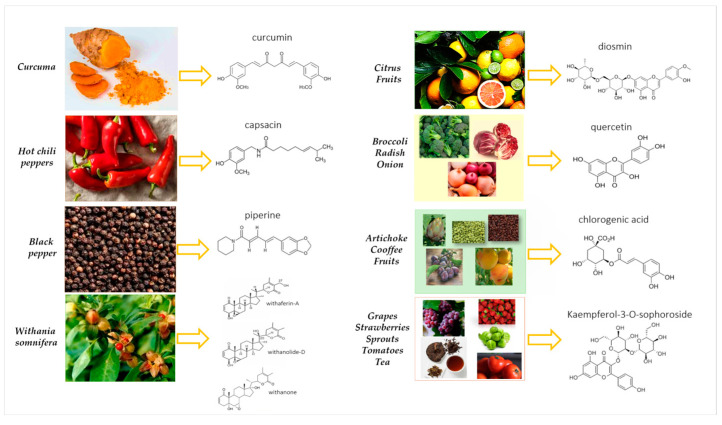
Polyphenols naturally present in fruits and vegetables that are considered effective in alleviating FM symptomatology thanks to their antinociceptive properties and anti-inflammatory activity.

In clinical studies focused on treating FM syndrome, a randomized, active-controlled trial was conducted to evaluate the analgesic and antinociceptive effects of *diosmin*, a natural flavone glycoside abundant in various species of citrus fruits. Interestingly, it was shown that diosmin can bind to opioid and D2 dopaminergic receptors and suppress peripheral pro-inflammatory cytokines, alleviating thermal and mechanical hyperalgesia in an in vivo experimental model [114]. In addition, in one study, the intravenous administration of diosmin (50 mg/kg, oral) for three days to 150 patients with radicular pain was compared to mannitol (81 g/kg/day) and dexamethasone (10 mg/day) administered to another group of 150 patients: only patients treated with diosmin experienced significant pain relief without adverse effects [115]. Interestingly, in a double-blind parallel-group clinical trial, patients with FM were randomized to receive either 15 mg of saffron (from *Crocus sativus* L.) or 30 mg of the drug duloxetine starting with one capsule per day in the first week, followed by two capsules per day from week two until the end of week eight. No significant difference was detected for any of the FM evaluating scales in terms of score changes from baseline to endpoint between the two treatment arms, indicating that the C*rocus sativus* natural extract and duloxetine had comparable efficacy in the treatment of FM symptoms [53,116,117]. Noteworthy, several plant extracts and natural compounds are currently utilized for their antinociceptive properties and potential to treat FM, and many phenolic compounds from medicinal plants are promising candidates for new natural analgesic drugs [118]. Numerous in vitro and in vivo studies have shown that many polyphenols possess the potential to fight against chronic fatigue as well as FM syndrome predominantly by counteracting oxidative stress and the inflammatory cascade [119,120], modulating the enzymatic activities involved in arachidonic acid metabolism (such as phospholipase A2 and COX) and arginine metabolism (such as NOS), as well as modulating the production of pro-inflammatory molecules [121]. Novel therapies are continuously developed using validated animal models. *Quercetin* found in various vegetables and fruits, such as berries, lovage, capers, cilantro, dill, apples, and onions, have analgesic activity attenuating hypersensitivity to pain through the inhibition of mTOR/p70S6K pathway-mediated changes in synaptic morphology and synaptic protein levels in the spinal dorsal horn neurons of db/db mice [122]. Other authors recently published the results obtained with quercetin derivatives of Tilia americana; an aqueous Tilia extract (TE) and its flavonoid fraction (FF) containing *rutin* and *isoquercitrin* were evaluated alone and/or combined with clinical drugs, such as Tramadol (TRA) and pramipexol (PRA). In this study, the potential effects of Tilia extract and FF administered with or without TRA and PRA were explored in reserpine-induced FM-type pain in rats, corroborating its presence at the central level by epifluorescence histological examination and receptor interaction by in silico analysis, confirming their presence in the brain. TE (10–100 mg/kg, i.p.) and FF (10–300 mg/kg, i.p.) produced significant and dose-dependent antihyperalgesic and antiallodynic effects equivalent to Tramadol (3–10 mg/kg, i.p.) or pramipexol administered subcutaneously (0.01–1 mg/kg) [123]. Like other flavonoids, *kaempferol* has interesting anti-inflammatory and antioxidant properties. Also known as kaempferol-3 or kaempferide, it is found naturally in tea, as well as in numerous vegetables and fruits, including beans, broccoli, cabbage, onions, grapes, strawberries, tomatoes, citrus fruits, Brussels sprouts, and apples. Interestingly, some glycosides of kaempferol (e.g., kaempferol 3-O-sophoroside) possess significant analgesic activity in the tail clip, tail flick, tail immersion, and acetic acid-induced writhing models, whereas baicalin has analgesic effects on several kinds of pain [124]. The analgesia potency of *hyperin* is approximately twenty times that of morphine, while luteolin presents effective analgesic activities for both acute and chronic pain management [125]. Notably, *fisetin* possesses potent antioxidant, antinociceptive, and neuroprotective activities [126]. *Chlorogenic acid* is a polyphenol that is most often present in plants, fruits, and vegetables, including peaches, prunes, potatoes, coffee, and beans. Coffee is the richest source of chlorogenic acid, which is formed by the esterification of caffeic acid and quinic acid. Noteworthy, chlorogenic acid was proven to be effective in alleviating streptozotocin-induced neuropathic pain through an antioxidative mechanism and downregulation of COX-2 expression [127]. *Hesperidin* is a well-known flavanone glycoside present in grapefruits and citrus fruits and this phytochemical possesses antioxidant and anti-inflammatory properties and is also a useful neuroprotective compound in the treatment of neuropathic pain [128]. Notably, an in vivo study found that hesperidin alone (five doses; 10–1000 mg/kg) or in combination with diosmin (10 and 100 mg/kg) led to a decrease in mechanical and thermal hyperalgesia in rats by binding to the D2 and opioid receptors [129]. Furthermore, hesperidin (100 mg/kg) was recently reported to have an antihyperalgesic effect in an in vivo experimental model and this phytochemical alleviated mechanical and thermal hyperalgesia through a decrease in the levels of cytokines such as TNF-α, IL-1β, and IL-6 [130]. Interestingly, an in vivo study demonstrated that naringenin’s analgesic and antioxidant activities are mediated by its ability to bind to TRPV1 receptors [131]. It was also shown that naringenin (10 mg/kg) suppressed mechanical allodynia and reduced the number of nociceptive responses and pro-inflammatory prostaglandin E2 levels in in vivo experimental models of nociception [132]. Natural molecules can interact with numerous molecular targets such as G-protein coupled receptors, neurotransmitters, ion channels, and inflammatory mediators. New targets of pain treatments, such as sigma receptors, D-amino acid oxidase, and stress receptors of the endoplasmic reticulum, have been considered [133]. The potential mechanisms of action of the many natural molecules cited here have already been reported in our previous article [134], where we extensively described several molecular pathways, including those related to oxidative stress and its association with fatigue in FM. These mechanisms are specifically reported in Table 2, which details various biological activities, such as the antioxidant and anti-inflammatory of polyphenols, as well as their ability to modulate molecular targets. In particular, the MAPK signaling pathway, which induces the inflammation process, stimulates microglial activation and pain sensitization via many signaling molecules such as chemokines, cytokines, and kinases regulated by the p38 protein. Phosphorylated p38 activates NF-kB, which triggers the release of additional inflammatory mediators, including cyclooxygenase-2 (COX-2), the brain-derived neurotrophic factor (BDNF), IL-6, TNF-α, and IL-1β. These mediators cause the sensitization of the central nervous system to pain signals and stimulate the development of FM syndrome. Moreover, some natural products like capsaicin (Figure 4) have been tested in preclinical and clinical studies, and their possible role in chronic pain management and the maintenance of physical and mental wellbeing has been demonstrated in patients with FM [58].

Certainly, in addition to administering nutrients that provide an energetic and trophic function for the muscles (Coenzyme Q10, B vitamins, carnitine, and magnesium), the use of antioxidants (vitamin C, E, A, polyphenols) and vitamin D has also had a beneficial effect on the symptoms of FM. For hundreds of years, plant extracts have been used to relieve musculoskeletal pain. Natural products represent a new possibility for the design of innovative analgesics and antinociceptive molecules to address pain-related conditions. Some natural molecules, for example, capsaicin, ginger, curcumin, and naringin [100,135], are being considered for FM therapy due to the numerous benefits displayed by these food-derived bioactive components towards FM-like symptoms. However, despite the use of a variety of dietary supplements and natural products with high nutritional value and the ability to bind to multiple molecular targets to reduce the signs and symptoms of neuropathic pain and FM, the number of preclinical and clinical studies set up to define their safety, efficacy and potential mechanisms is still low. Therefore, further investigations are required to assess the therapeutic value of bioactive compounds for patients with FM. There is a need for new prevention and treatment strategies for FM based on the use of bioavailable nutraceuticals and/or phytochemicals providing health benefits, especially considering the impact of this condition on overall health, as evidenced by population-based data [136]. *Piperine*, a component of black pepper extract, also shows significant analgesic and neuroprotective efficacy with potential additive efficacy in FM [137,138].

## 6. Role of Gut Microbiota

Among all these studies focused on diagnostic strategies to detect changes in patients with FM compared to healthy individuals, some researchers have highlighted the overlap in symptomatology between FM and irritable bowel syndrome (IBS). IBS is one of the most prevalent chronic gastrointestinal diseases and is often associated with alterations in the gut microbiota of patients who have recurrent abdominal pain, gas, diarrhea, constipation, gastroesophageal reflux, and esophageal hypersensitivity. Although the physiological mechanism of these two diseases is not completely understood, some authors suggest a common etiology between FM and IBS, which frequently coexist specifically among women. An important relationship has recently been reported between the prevalence and severity of disorders related to IBS and gut–brain axis disruptions in women living with FM pain. These included issues such as headaches, migraines, sleep disturbances, and reduced quality of life. Researchers have explained how improvements in one area, such as better sleep or reduced IBS symptoms, may lead to reduced pain and an overall improvement in wellbeing [139]. Similarities in microbiota composition between FM and IBS patients have also been demonstrated, and it has been suggested that a link between the gut microbiota and the central nervous system exists, which may, in turn, control the bowel’s microbiota constituents. Interestingly, recent evidence has shown that gut microbiota manipulations can be successfully used as a therapeutic approach both for IBS and FM in patients. These interventions have been shown to alleviate intestinal symptoms, reduce pain, and improve physical rehabilitation outcomes. Both FM and IBS are more prevalent in women and share characteristics such as sympathetic nervous system dysfunction and central sensitization. Additionally, many of the clinical aspects of IBS, such as extra-intestinal comorbidities, insomnia, and chronic fatigue, are often associated with FM syndrome [140,141,142]. Qualitative and quantitative changes in the gut microbiota, which influence gut–brain axis communication, could create unfavorable conditions contributing to the development of FM. An imbalance in gut microbial populations negatively affects gut homeostasis and might cause inappropriate activity of the gut–brain axis [143] as well as impairments in the central processing of sensory inputs. Alterations in this axis have been associated with gastrointestinal syndromes, as highlighted in recent research [144], and changes in gut microbiota composition have also been reported in patients with FM [145]. This connection has attracted attention among researchers, as the gut microbiota is thought to play a role in the regulation of chronic pain. The gut microbiota is known to contribute to central sensitization in chronic pain and inflammatory diseases such as endometriosis by regulating microglia, astrocytes, and immune cells [146]. If alterations in gut microbiota may affect the gut–brain axis [147], it is also likely that dysbiosis might play a role in FM pathogenesis by altering the perception and processing of painful stimuli. Studies analyzing gut microbiota in patients with FM have reported altered bacterial compositions. An enrichment or a depletion of specific species of bacteria could affect the severity of symptoms; in particular, bacterial species belonging to the families of *Lachnospiraceae* and *Ruminococcaceae* as well as to *Eubacterium* and *Bifidobacterium* genera showed a lower abundance in the intestinal microbiota of patients with FM, while the Rikenellaceae family and many species belonging to the *Clostridia* class were overrepresented [145]. Therefore, it is interesting to note the important role of some microbiota bacteria; for example, those of the Bifidobacterium genus participate in the metabolism of neurotransmitters by synthesizing gamma amino butyric acid (GABA) from glutamate. The gut microbiota also produces a range of other neurotransmitters and neurotrophic factors, such as dopamine, noradrenaline, serotonin, acetylcholine, and histamine, which influence signaling between the gut (enteric microbiota) and the central nervous system. The brain itself acts on enteric microbiota via changes in gastrointestinal motility, permeability, and secretion [148]. As already reported, GABA is the most important inhibitory neurotransmitter in the CNS, reducing pain perception and transmission by nociceptive neurons by inducing neuron hyperpolarization and increasing the excitability threshold. On the contrary, glutamate, a major excitatory neurotransmitter, promotes pain sensitization [10,149]. For this reason, a reduced presence of *Bifidobacteria*, which is able to produce GABA, can cause an imbalance in the ratio between GABA and glutamate in favor of the latter [150]. Consequently, the heightened and widespread pain sensitivity observed in patients with FM may be associated with the diminished ability of the gut microbiota to produce GABA. This deficiency, together with increased gut barrier permeability, could lead to the systemic accumulation of glutamate and extensive excitation of nociceptor neurons, further amplifying pain signals. Additionally, many bacterial species whose abundance is altered in patients with FM are involved in the metabolism of short-chain fatty acids (such as acetate, propionate, and butyrate), which is fundamental for maintaining intestinal barrier integrity. They promote the expression of mucins, antimicrobial peptides, and tight junction proteins, supporting gut health [151]. Of note, a recent clinical trial started in 2024 with the involvement of a group of 80 women (half of which will receive the active treatment, while the other half of participants will receive a sham treatment) diagnosed with severe FM. Fecal microbiome transplantation from healthy donors to individuals with FM appears to improve pain, fatigue, and sleep problems. This clinical study is still in progress, and the symptoms of the female participants, alongside changes in certain metabolites in their blood and stools, will be documented (https://clinicaltrials.gov/study/NCT06424041?cond=Fibromyalgia&intr=microbiota&rank=1 (accessed on 21 May 2024)). The results of this treatment could offer a significant advantage in managing FM, providing new insights into the role of the human gut microbiota in the pathogenesis of pain and also offering the opportunity to identify it as a potential target for analgesic therapies [152].

## 7. Conclusions

Fibromyalgia (FM) is typically characterized by persistent widespread pain associated with reduced pressure pain thresholds, hyperalgesia, and allodynia, as well as altered sleep, fatigue, and mood disturbances, all of which have serious effects on patients’ quality of life. Patients with FM treated with milnacipran, serotonin, and norepinephrine reuptake inhibitors show a reduction in pain sensitivity and a parallel increase in activity in brain regions implicated in descending pain inhibitory pathways compared to patients treated with placebos. To date, pharmacological treatment strategies for FM include the use of drugs such as antidepressants, calcium channel modulators, muscle relaxants, and analgesics (e.g., duloxetine, pregabalin, and tramadol for pain and amitriptyline, cyclobenzaprine, and pregabalin for sleep disturbance) although they have shown limited efficacy and therapeutic adherence. FM remains a complex condition to treat and is an important challenge for modern medicine, as current treatments are often ineffective and associated with numerous side effects, making the need for new treatments increasingly urgent. Increasingly, the management of FM includes not only pharmacological treatments but also non-pharmacological therapies based on natural molecules. In fact, both clinical and preclinical studies have investigated the analgesic potential of natural substances, including plant extracts, for the management of FM. The successful clinical use of these products, with no significant reports of adverse effects, emphasizes the advancements made in utilizing natural products, such as polyphenols, for the treatment and management of FM. Dietary approaches that reduce processed foods and refined carbohydrates but are instead rich with plant-based foods and natural molecules such as polyphenols may help manage FM symptoms. Notably, researchers have also made significant strides in developing innovative phytochemical delivery strategies, including those inspired by the red blood cell-based biomimetic system, to preserve the bioactive properties of natural molecules like polyphenols.

## Figures and Tables

**Table 1:** Outline of miRNAs linked to FM. * and ** see comments in Characterization column.

miRNA Identity		Characterization
miR-374		Downregulated in the plasma of individuals with temporomandibular disorder and fibromyalgia syndrome. [22]
miR-23a	miR-152	Involved in multiple crucial pathological processes for FM and/or ME/CFS. [21]
miR-103	miR-320	
hsa-miR-29a-3p **		* Upregulated in both FM and osteoporosis (OP). ** Downregulated in both FM and OP. [23]
hsa-miR-9-(3p or 5p) *		
hsa-miR-128-(3p) *		
hsa-335-5p *		
hsa-miR-1-(3p) **		
hsa-let-7a-(3p or 5p) **		
hsa-miR-328-3p **		
hsa-miR-1-3p		Detection of miRNA-related SNPs in a small FM cohort. [19]
hsa-miR-130a-3p		
microRNA-320a		Increased levels in FM individuals. * Targets bone morphogenic protein receptor 2 and interleukin 6 in FM individuals. [15,21]
microRNA-320b		
microRNA-142-3p		
microRNA-182-5p *		
several circulating miRNAs		The expression of circulating miRNAs can differentiate between different disorders (ME/CFS, FM, and ME/CFS + FM). [20]
microRNA-320a		Significantly upregulated in patients with FM. Impact of the severity of symptoms but not related to the cerebral processing of pain. [15]
hsa-miR-182-5p		Upregulated in women with FMS compared to healthy controls. The mRNA of target genes BMPR2 and IL6ST were downregulated in FMS. [24]
miR-320a		The content of these miRNAs in the plasma of female patients with FMS is correlated to their general health and functional status and mental symptom score. [16,17]
miR-320b		
miR-142-3p		
Hsa-mir-367 *	Hsa-mir-148a **	* Upregulated in patients with FM. ** Downregulated in patients with FM. [25]
Hsa-mir-646 *	Hsa-mir-338-3p **	
Hsa-mir-876-3p *	Hsa-mir-451 **	
Hsa-mir-548z *	Hsa-mir-145 **	
Hsa-mir-3164 *	Hsa-mir-143 **	
miR130a-3p		Lower gene expression in patients with FM. * Related to adaptive coping in a cluster of patients with FM. [26]
miR103a-3p *		
miR107 *		
miR-let-7d		miR-let-7d and its downstream target insulin-like growth factor-1 receptor is aberrantly expressed in the skin of patients with FMS with small nerve fiber impairment. [27]
miR-34a-5p		Migraine attacks were associated with an acute upregulation in serum miR-34a-5p and miR-382-5p expression. [28]
miR-29c-5p		
miR-382-5p		
miR-26b-3p		
hsa-miR223-3p		Marked downregulation in PBMCs in patients with FM. No significant correlations between miRNA inhibition and FM cardinal symptoms were identified. The five strikingly downregulated miRNAs are proposed as biomarkers of FM. [29]
hsa-miR451a		
hsa-miR338-3p		
hsa-miR143-3p		
hsa-miR145-5p		
miR-107	miR-103a-3p	Lower expression in FM versus healthy women, except for miR-320a (* higher expression in FM). miR-103a-3p is correlated with pain and sleep quantity in FM. miR-320a and miR-374b-5p are correlated inversely with pain threshold. miR-30b-5p correlates with sleep quantity Let-7a-5p is associated with sleep symptoms. [30]
let-7a-5p	miR-142-3p	
miR-30b-5p	miR-374b-5p	
miR-151a-5p	miR-320a *	
miR-21-5p	miR-125b-5p	Lower expression in cerebrospinal fluid of patients with FM. [31,32]
	miR-23a-3p	
miR-145-5p	miR-23b-3p	
miR-29a-3p	miR-195-5p	
miR-99b-5p	miR-223-3p	

**Table 2:** FM assessment reports and examples of physical conditions associated with this syndrome.

Self-Report Forms for Assessing Patients’ Pain	Conditions Co-Existing with Fibromyalgia
❖ Checklist of current symptoms ❖ Scales for helplessness and cognitive performance ❖ Multidimensional Health Assessment Questionnaire (MDHAQ) ❖ Fibromyalgia Impact Questionnaire (FIQ) ❖ Generalized Anxiety Disorder-7 questionnaire (GAD-7) ❖ Physician Health Questionnaire-9 for depression (PHQ-9) ❖ The Mood Disorder Questionnaire to screen for bipolar disease (MDQ)	Musculoskeletal pain
	Stiffness of the joints
	Allodynia and hyperalgesia
	Chronic fatigue syndrome
	Temporomandibular joint disorders
	Migraine and other types of headaches
	Peripheral paresthesia (tingling, cramps)
	Sleep disruption (insomnia)
	Unexplained symptomatology such as anxiety
	Depression
